# Consequences of induced brassinosteroid deficiency in Arabidopsis leaves

**DOI:** 10.1186/s12870-014-0309-0

**Published:** 2014-11-18

**Authors:** Florian Schröder, Janina Lisso, Toshihiro Obata, Alexander Erban, Eugenia Maximova, Patrick Giavalisco, Joachim Kopka, Alisdair R Fernie, Lothar Willmitzer, Carsten Müssig

**Affiliations:** University of Potsdam, c/o Max Planck Institute of Molecular Plant Physiology, Am Mühlenberg 1, 14476 Potsdam-Golm, Germany; Max Planck Institute of Molecular Plant Physiology, Am Mühlenberg 1, 14476 Potsdam-Golm, Germany

**Keywords:** Brassinosteroids, Arabidopsis, Tricarboxylic acid cycle, Biomass, Cell expansion, Growth

## Abstract

**Background:**

The identification of brassinosteroid (BR) deficient and BR insensitive mutants provided conclusive evidence that BR is a potent growth-promoting phytohormone. Arabidopsis mutants are characterized by a compact rosette structure, decreased plant height and reduced root system, delayed development, and reduced fertility. Cell expansion, cell division, and multiple developmental processes depend on BR. The molecular and physiological basis of BR action is diverse. The BR signalling pathway controls the activity of transcription factors, and numerous BR responsive genes have been identified. The analysis of dwarf mutants, however, may to some extent reveal phenotypic changes that are an effect of the altered morphology and physiology. This restriction holds particularly true for the analysis of established organs such as rosette leaves.

**Results:**

In this study, the mode of BR action was analysed in established leaves by means of two approaches. First, an inhibitor of BR biosynthesis (brassinazole) was applied to 21-day-old wild-type plants. Secondly, BR complementation of BR deficient plants, namely *CPD* (*constitutive photomorphogenic dwarf*)-antisense and *cbb1* (*cabbage1*) mutant plants was stopped after 21 days. BR action in established leaves is associated with stimulated cell expansion, an increase in leaf index, starch accumulation, enhanced CO_2_ release by the tricarboxylic acid cycle, and increased biomass production. Cell number and protein content were barely affected.

**Conclusion:**

Previous analysis of BR promoted growth focused on genomic effects. However, the link between growth and changes in gene expression patterns barely provided clues to the physiological and metabolic basis of growth. Our study analysed comprehensive metabolic data sets of leaves with altered BR levels. The data suggest that BR promoted growth may depend on the increased provision and use of carbohydrates and energy. BR may stimulate both anabolic and catabolic pathways.

**Electronic supplementary material:**

The online version of this article (doi:10.1186/s12870-014-0309-0) contains supplementary material, which is available to authorized users.

## Background

Brassinolide (BL) was identified in 1979 through its ability to promote internode growth [1]. Since then, the growth-promoting effect has been studied in hundreds of articles. Excised tissues (e.g. hypocotyl, epicotyl, cotyledons, internodes, leaves, and roots), protoplasts, cell suspension cultures, intact seedlings, and whole plants were subjected to brassinosteroid (BR) treatments, and in all cases BR had the potential to stimulate growth [2]. A large number of BR deficient and BR insensitive mutants in *Arabidopsis thaliana* and in crops such as rice, tomato, and pea were identified [3]. These mutants are generally dwarfed and exhibit rounded, dark green leaves, delayed flowering, reduced male fertility and seed set, and delayed senescence. Further proven roles of BR include the control of xylem formation [4,5], stomata development [6-8], and further developmental processes [9-12]. Numerous studies analysed gene expression patterns upon BR treatment and BR deficiency. However, these studies barely clarified the metabolic and physiological basis of BR dependent growth because the precise functions of isoenzymes, cell wall proteins, and other factors often remains obscure.

BR plays non redundant roles since it is not possible to complement BR mutants with other phytohormones or their antagonists [13]. Overexpression of the major BR receptor, BRI1 (BRASSINOSTEROID-INSENSITIVE 1), stimulated growth. However, the underlying changes at transcript and metabolite level are largely different from other growth-stimulating pathways [14]. The most prominent direct BR effect is the modification of gene expression patterns [15]. Transcription factors such as BES1 (*bri1*-EMS-suppressor 1) and BZR1 (Brassinazole-resistant 1) regulate BR responsive genes [16-19]. The physiological mechanisms underlying BR promoted growth appear to be manifold, and depend on the tissue and developmental stage [2]. They include the control of aquaporin activity and water movement across membranes [20], cytoskeleton organisation [21-23], and the modification of mechanical cell wall properties [24,25]. A few studies in Arabidopsis and crops addressed the influence of BR on primary metabolism. The focus of these studies was on photosynthesis and sink strength, and specific enzyme activities or metabolites were measured. An early article showed that BR stimulates CO_2_ assimilation in wheat [26]. This finding was confirmed in other plants such as cucumber [27] and rice [28]. In Arabidopsis, expression of a mutated BRI1 receptor (Y831F) enhanced shoot growth and conferred elevated photosynthetic rates and starch levels at the end of the light period [29]. Rubisco activity and regeneration of Ribulose-1,5-bisphosphate are the most important limiting factors of photosynthesis under natural conditions. Positive effects of BR on Rubisco activity have been shown [26,27]. Yu *et al.* [27] also postulated a positive effect on Ribulose-1,5-bisphosphate-regeneration. Enhanced photosynthesis correlated with an increase of soluble sugar and starch content and parallel enhancement of fresh and dry weights. In line with these data, BR deficient Arabidopsis mutants showed drastically reduced CO_2_ assimilation rates, reduced starch levels, a tendency to reduced sucrose levels, and reduced biomass accumulation [30].

In addition to source efficiency, BR also increases sink strength. The tomato *d*^*x*^ mutant produces bioactive BR in fruits but not in the shoot, and provides an option to dissect BR dependent processes in fruits and shoots. Dry weight and starch levels of *d*^*x*^ fruits were significantly reduced [31]. BR application to leaves partly normalized metabolic changes in *d*^*x*^ fruits suggesting that shoot-derived BR dependent factors are required for proper fruit metabolism. Previous research emphasized the relevance of BR for invertase activity in the growing zone of tomato hypocotyls [32]. Thus, several reports demonstrated the requirement of BR for source efficiency and sink strength.

In this study, induced BR deficiency was analysed in Arabidopsis rosette leaves by means of complementary time-series experiments. First, BR deficient plants were complemented by exogenous BR. Subsequent omission of BR treatment caused BR deficiency. Secondly, brassinazole (BRZ) was applied to wild-type plants. BRZ binds to the DWF4 enzyme and specifically blocks BR biosynthesis at the C-22 hydroxylation step [33]. BR dependent growth of established leaves is associated with elevated starch levels, higher metabolic flux through the tricarboxylic acid (TCA) cycle, and increased cell expansion and biomass production.

## Results

### Time course experiments for the analysis of morphological and biochemical consequences of BR deficiency

The analysis of BR deficient or BR insensitive mutants is complicated by the severe dwarfism and major morphological changes [3]. The use of mutants with mild phenotypic changes such as *cbb1*/*dwf1* (*cabbage1/dwarf1*) [13,34] can alleviate that difficulty. The known alleles result in milder phenotypic changes in comparison to *det2* (*de-etiolated2*), *cpd* (*constitutive photomorphogenic dwarf*), *dwf4* (*dwarf4*), and other biosynthetic mutants. The *cbb1*/*dwf1* mutants are presumably able to produce unusual bioactive BR as a consequence of the accumulation of precursors and display altered BR responses [34,35].

**Figure 1:**
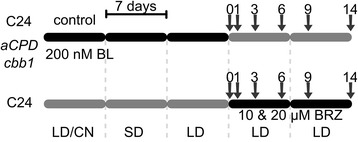
**Design of time series experiments.** Plants were grown on soil for 5 weeks in controlled growth chambers under the specified conditions. Black bars indicate a treatment of the plants with brassinolide (BL) or brassinazole (BRZ). Samples were taken at day 0 (21 days after sowing), 1, 3, 6, 9 and 14. LD, long day; SD, short day; CN, cold night.

In order to start the analysis of BR deficiency symptoms with morphologically intact plants, two complementary sets of time course experiments were performed (Figure 1). During the time course experiments, plants were grown in parallel in a randomized manner in a controlled growth chamber (see Methods for details). The time course experiments were performed three times each, resulting in a total of six independent experiments.

**Figure 2:**
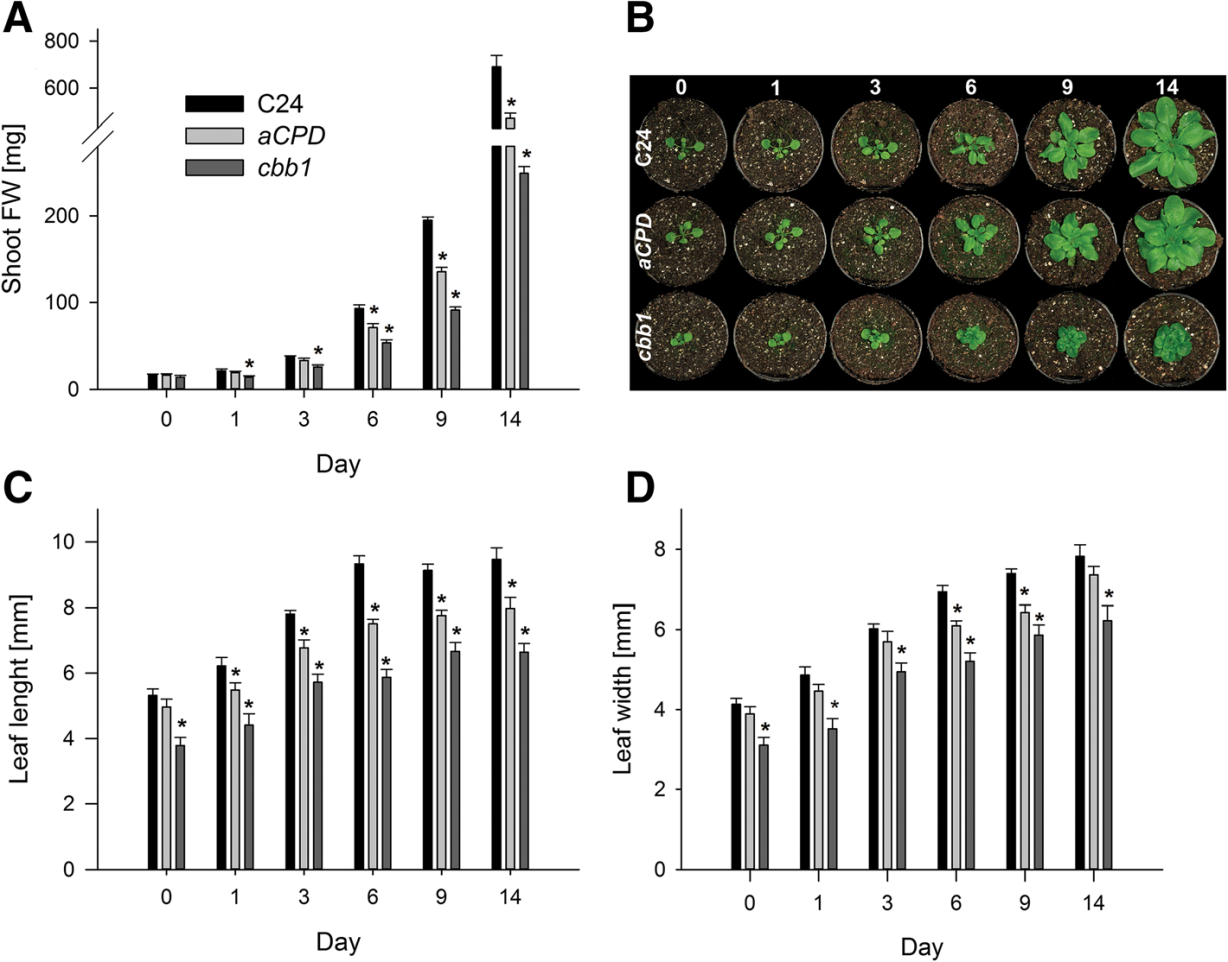
**Growth parameters of** ***CPD*** **-antisense and** ***cbb1*** **plants in comparison to the wild type.** Wild-type (C24), *CPD-*antisense, and *cbb1* plants were grown as described in Figure 1. **A**, Shoot fresh weight. **B**, Representative plants at day 0, 1, 3, 6, 9, and 14. **C**, Length of rosette leaves three and four. **D**, Width of rosette leaves three and four. Data are given as mean ± SE (n =10 plants). Values denoted with an asterisk are significantly different from the wild type (t test, P <0.05).

The first approach used BR deficient mutants. *CPD*-antisense (*aCPD*) plants and the BR-deficient *cbb1/dwf1-6* mutant [13,30] were treated with 200 nM brassinolide (BL) for three weeks. Wild-type plants were grown in parallel and were simultaneously treated with a control solution. The control solution was identical to the BR solution apart from the addition of BR (for details see Methods). BR supplementation fully normalized the morphology and biomass production of *CPD*-antisense plants. The *CPD*-antisense plants were nearly indistinguishable from the wild type. The growth defect of *cbb1* plants was partly complemented by exogenous BR (Figure 2, day 0). Fresh weight of 21-day-old *cbb1* shoots was identical to the wild type. However, leaf length and width were diminished in comparison to the wild type, leaves were more erect and had a slightly crinkled surface, and rosettes appeared compact. Thus, exogenous BR could not fully substitute for endogenous BR. After three weeks, BR treatment was stopped (day 0). The *CPD*-antisense and *cbb1* plants started to run into BR deficiency or pronounced BR deficiency, respectively. At this point the sampling began. Samples for biochemical analysis were taken at day 0, 1, 3, 6, 9 and 14 after the respective treatment was stopped. Under the applied conditions, plants were in the vegetative phase during the complete experiments and did not start bolting.

**Figure 3:**
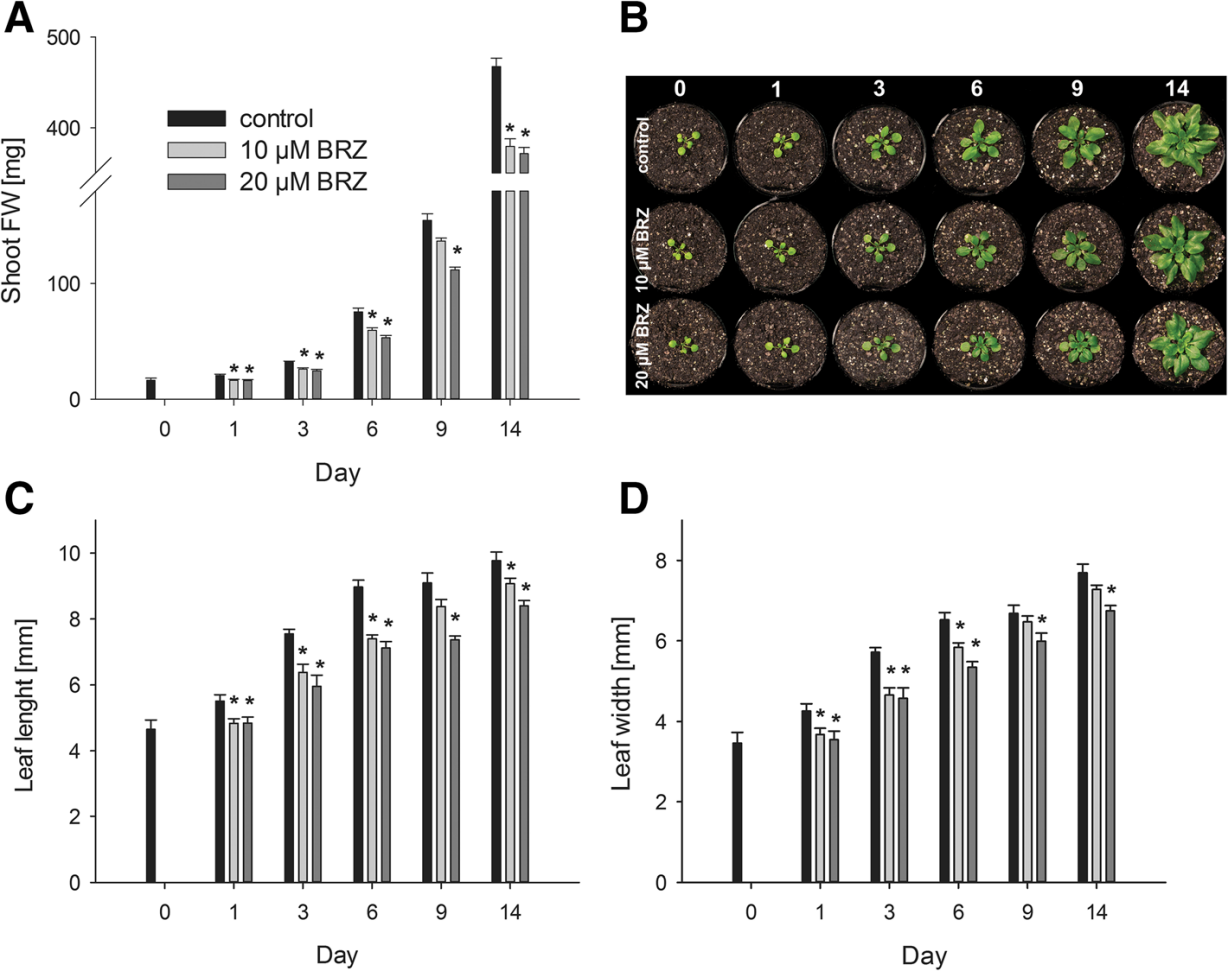
**Growth parameters of BRZ treated plants in comparison to mock treated plants.** Wild-type plants were grown and treated as described in Figure 1. **A**, Shoot fresh weight. **B**, Representative plants at day 0, 1, 3, 6, 9, and 14. **C**, Length of rosette leaves three and four. **D**, Width of rosette leaves three and four. Data are given as mean ± SE (n =10 plants). Values denoted with an asterisk are significantly different from the wild type (t test, P <0.05).

The second approach used wild-type plants (C24) that were grown for three weeks without any treatment (Figure 3, day 0). Subsequently, plants were treated with 10 μM BRZ, 20 μM BRZ, or control solution. Lower concentrations such as 1 to 5 μM BRZ have previously been applied in synthetic growth medium (e.g. [36,37]) but were inapplicable in our time course experiments since they induced only minor growth effects in soil-grown plants. The necessity for higher BRZ concentrations may reflect weaker uptake by leaves through a functional epidermis. After the onset of BRZ application (day 0), samples for biochemical analysis were taken at the same points in time as described above (i.e. day 1, 3, 6, 9, and 14).

The parallel analysis of *CPD*-antisense, *cbb1*, and BRZ treated wild-type plants allows avoiding genotype- or treatment-specific limitations during the evaluation of BR deficiency.

### Elevated *CPD* and *DWF4* transcript levels indicate emerging BR deficiency

**Figure 4:**
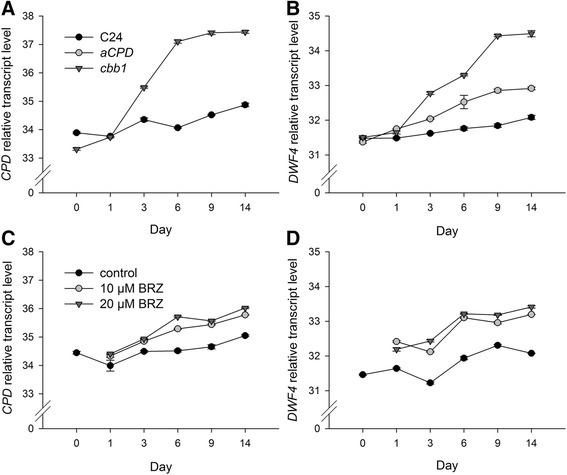
**Quantitative RT-PCR analysis of** ***CPD*** **and** ***DWF4*** **transcript levels.** Plants were grown and harvested as described in Figure 1. **A**, Relative *CPD* transcript levels in wild-type (C24) and *cbb1* plants. **B**, Relative *DWF4* transcript levels in wild-type, *CPD-*antisense, and *cbb1* plants. **C**, Relative *CPD* transcript levels in BRZ treated plants. **D**, Relative *DWF4* transcript levels in BRZ treated plants. *eIF1a* CT values were subtracted from respective CT values of the gene of interest resulting in dCT. Subsequently, differences were subtracted from an arbitrary value (i.e. 40). Higher numbers indicate higher transcript levels. A difference of one unit indicates a fold change of two. Data are given as mean ± SE of gene of interest in three technical replicates. The data shown are from one experiment representative of three independent biological replicates.

The *CPD* [38] and *DWF4* [39] genes encode enzymes involved in BR biosynthesis. The expression of these genes is negatively associated with endogenous BR levels. High transcript levels indicate low BR levels and vice versa [40]. *CPD* and *DWF4* transcript levels were analysed by means of quantitative RT-PCR (Figure 4).

Unchanged transcript levels one day after the BR supplementation was stopped or after the BRZ treatment was started may indicate the presence of remaining BR or a time lag in the induction of BR biosynthetic genes. Stronger differences were observed from day 3 onwards. *CPD* transcript levels in *CPD*-antisense plants were previously described [30]. Due to incomplete *CPD* gene repression, the phenotypic changes of *CPD*-antisense plants are considerably milder in comparison to the *cpd*/*cbb3* knock-out mutant and other BR deficient mutants such as *cbb1*/*dwf1-6* [13,38]. Stronger *DWF4* expression in the *cbb1* mutant in comparison to the *CPD*-antisense plants corresponds to the observed growth defect (Figure 4A and B). Only minor differences were detected between plants treated with different concentrations of BRZ. Application of 10 μM BRZ induced *CPD* and *DWF4* expression nearly as effectively as 20 μM BRZ (Figure 4C and D).

### Induced BR deficiency impairs leaf expansion

Significant differences in shoot fresh weight and length of rosette leaves three and four developed after one day in *cbb1* plants (Figure 2A and C). Both growth parameters of *CPD*-antisense plants became significantly different from the wild type after six days and one day, respectively (Figure 2A and C). At the end of the analysed period (day 14), *cbb1* and *CPD*-antisense plants had 36% and 68% of the wild-type fresh weight, respectively (Figure 2A). BR deficiency caused a less pronounced effect on leaf width (Figure 2D). The resulting decrease in the leaf index (i.e. more roundish leaves; [41]) is a well described feature of BR deficient plants (e.g. [42]). BRZ treated plants exhibited a small reduction in the shoot fresh weight at day 1 (Figure 3A). The biomass difference to control plants increased over time. Leaf length and leaf width were reduced. Similarly to the *CPD*-antisense and *cbb1* plants, leaf width was less affected than leaf length (Figure 3C and D).

**Figure 5:**
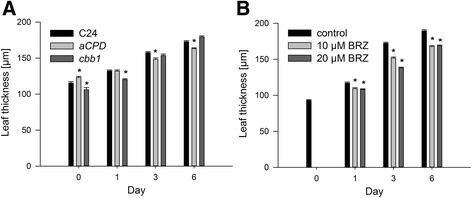
**Leaf thickness.** Plants were grown and harvested as described in Figure 1. Thickness of the leaves three and four was measured using transversal sections. Values are given as mean ± SE. 20 leaves were analysed per point in time. Values denoted with an asterisk are significantly different from the wild type (C24) or control (t test, P <0.05). **A**, Leaf thickness of wild-type (C24), *CPD-*antisense, and *cbb1* plants. **B**, Leaf thickness of BRZ treated plants.

Leaf thickness depends on the BR level and genotype. For example, BR deficient mutants such as *det2* exhibited an increased leaf thickness. Low concentrations of exogenous BR decreased leaf thickness of *det2* and wild-type plants. Higher concentrations caused an increase of leaf thickness in the wild type [43]. In this study, leaf thickness of *CPD*-antisense plants and BRZ treated plants displayed diminished enhancement from day 0 to day 6. In contrast, leaf thickness of *cbb1* plants increased more during that period (Figure 5).

Leaves five and six were analysed in parallel at day 3 and day 6. Similar effects on leaf length, leaf width, and leaf thickness were observed (Additional file 1: Figures S1 and S2, A-C).

### Reduced growth is mainly due to reduced cell size

Smaller leaf size of BR deficient plants could be based on impaired cell expansion, cell proliferation, or a combination of both. Previous analyses of BR mutants revealed effects on both cell proliferation and cell expansion. BR deficient mutants such as *dwf1*, *det2* [42] and *cpd* [44] are characterized by reduced cell division rates and reduced cell expansion.

**Figure 6:**
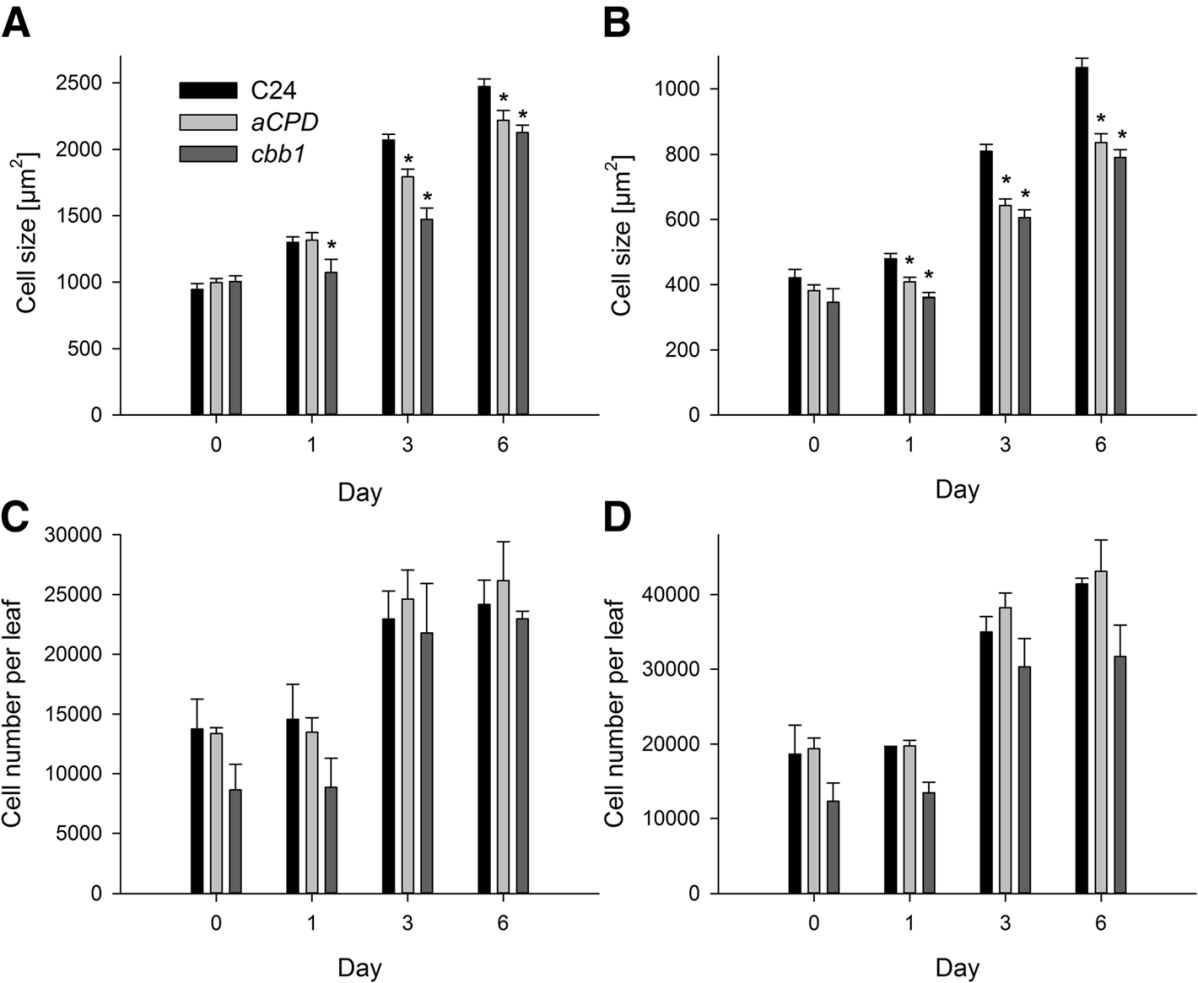
**Cell sizes and cell numbers of rosette leaves of** ***CPD*** **-antisense and** ***cbb1*** **plants.** Wild-type (C24), *CPD*-antisense, and *cbb1* plants were grown and harvested as described in Figure 1. Cell sizes were measured using transversal sections of rosette leaves three and four. Cell numbers were calculated from the respective other half of the same leaf. Data are given as mean ± SE. 20 leaves were analysed per point in time. Values denoted with an asterisk are significantly different from the wild type (t test, P <0.05). **A**, Area of palisade cells. **B**, Area of spongy parenchyma cells. **C**, Palisade cells per leaf. **D**, Spongy parenchyma cells per leaf.

**Figure 7:**
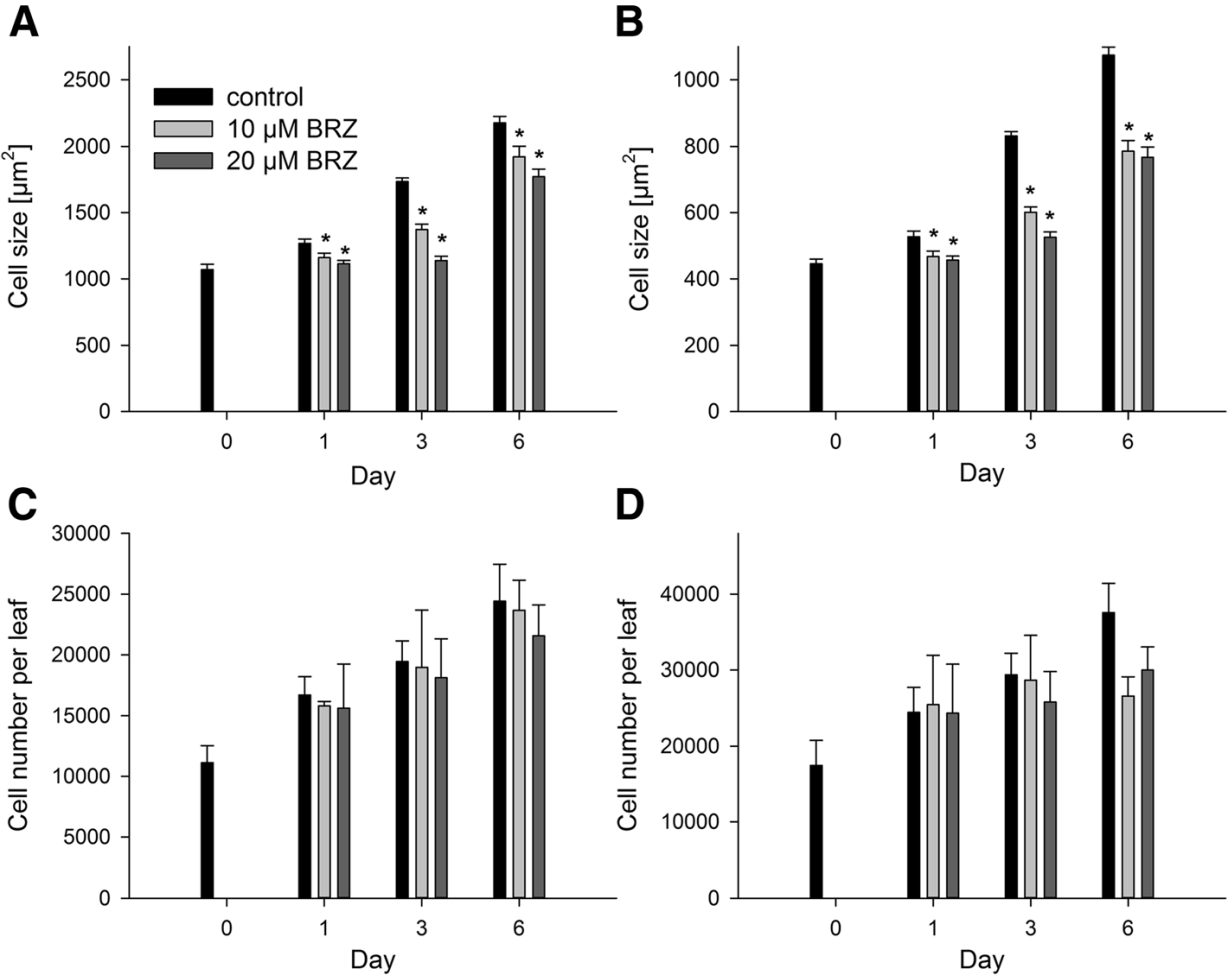
**Cell sizes and cell numbers of rosette leaves of BRZ treated plants.** BRZ treated plants were grown and harvested as described in Figure 1. Cell sizes were measured using transversal sections of rosette leaves three and four, and cell numbers were calculated from the respective other half of the same leaf. Data are given as mean ± SE. 20 leaves were analysed per point in time. Values denoted with an asterisk are significantly different from the wild type (t test, P <0.05). **A**, Area of palisade cells. **B**, Area of spongy parenchyma cells. **C**, Palisade cells per leaf. **D**, Spongy parenchyma cells per leaf.

In this study, similar size of palisade and spongy parenchyma cells in *CPD*-antisense, *cbb1*, and wild-type plants were observed at day 0 (Figure 6A and B), suggesting that the previous BR application normalized cell expansion. Later on, palisade and spongy parenchyma cells of *CPD*-antisense and *cbb1* plants became smaller in comparison to the wild type (Figure 6A and B). Similar results were obtained for younger leaves (Additional file 1: Figure S1, D and E). Effects on cell number were less evident. The *cbb1* leaves exhibited a tendency towards lower cell numbers, indicating an incomplete normalization of cell division rates by the previous BR treatment. In contrast, *CPD*-antisense plants were identical to the wild type at day 0 and later (Figure 6C and D; Additional file 1: Figure S1, F-H; Additional file 1: Figure S3A). Application of BRZ to the wild type reduced cell sizes (Figure 7A and B; Additional file 1: Figure S2, D and E), but did not significantly reduce cell numbers (Figure 7C and D; Additional file 1: Figure S2, F-H).

### Reduced starch and unchanged protein levels in BR deficient plants

**Figure 8:**
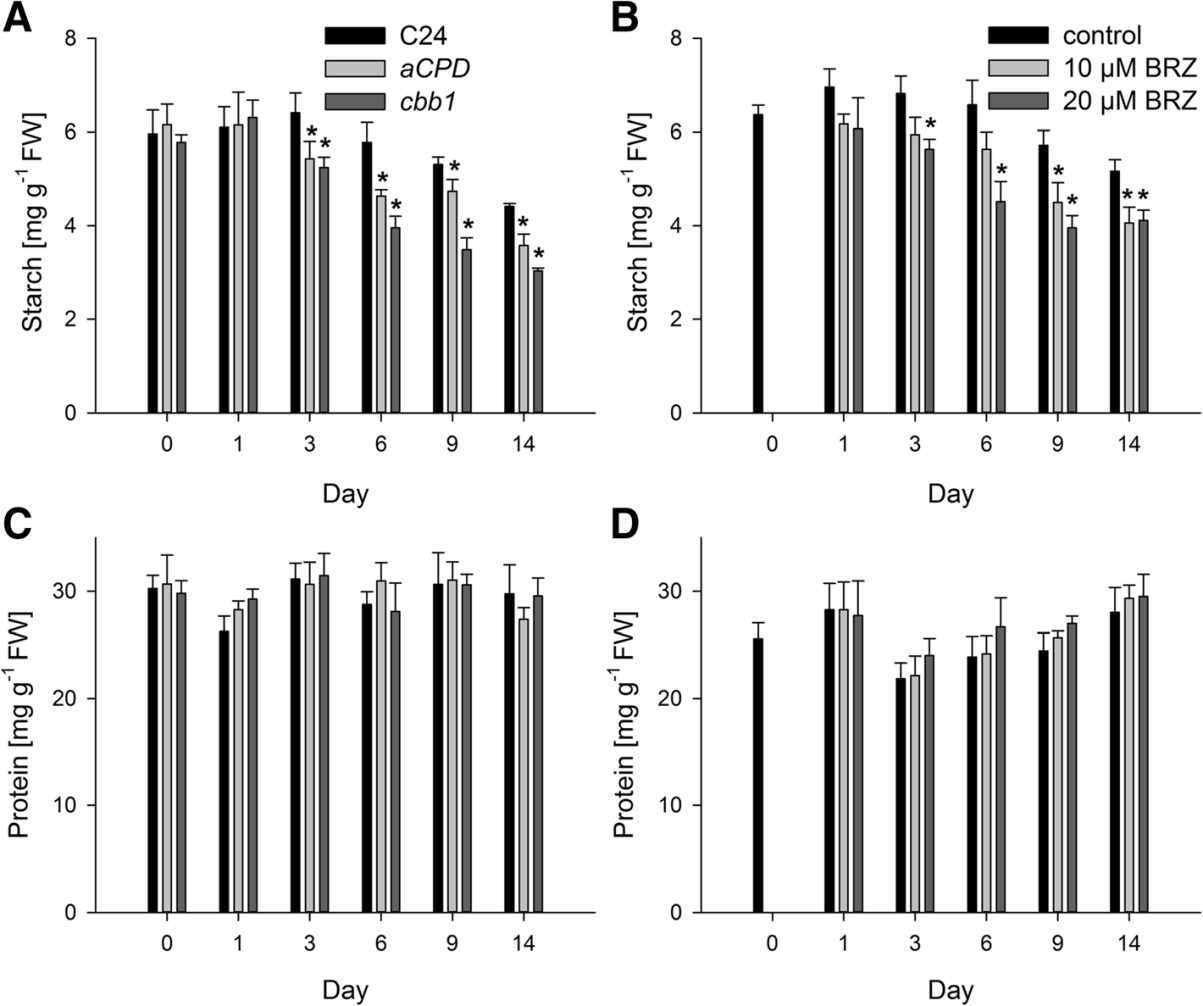
**Starch and protein levels.** Plants were harvested at the middle of the light period. Data are given as mean ± SE (n =3 pools of 10 plants). Values denoted with an asterisk are significantly different from the wild type or control (t test, P <0.05). **A**, Starch levels of wild-type (C24), *CPD*-antisense, and *cbb1* plants. **B**, Starch levels of BRZ treated plants. **C**, Protein levels of C24, *CPD*-antisense, and *cbb1* plants. **D**, Protein levels of BRZ treated plants.

**Figure 9:**
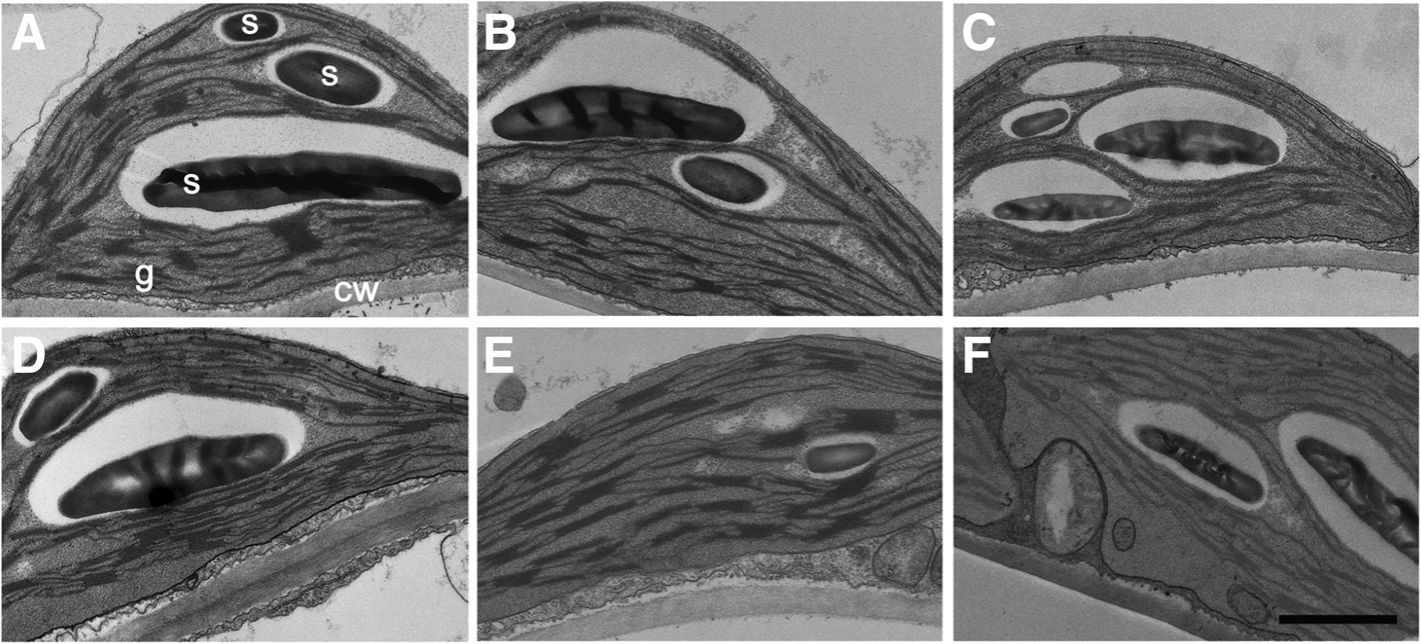
**Transmission electron microscopy of plastids in leaf three of** ***CPD*** **-antisense and** ***cbb1*** **plants.** Plants were grown and harvested as described in Figure 1 at the middle of the light period. **A**, Wild type (C24) at day 3. **B**, *CPD-*antisense at day 3. **C**, *cbb1* at day 3. **D**, C24 at day 6. **E**, *CPD-*antisense at day 6. **F**, *cbb1* at day 6. Subcellular structures are exemplarily indicated in **A**; cw, cell wall; g, granum (stack of thylakoids); s, starch granule; bar: 1 μm.

**Figure 10:**
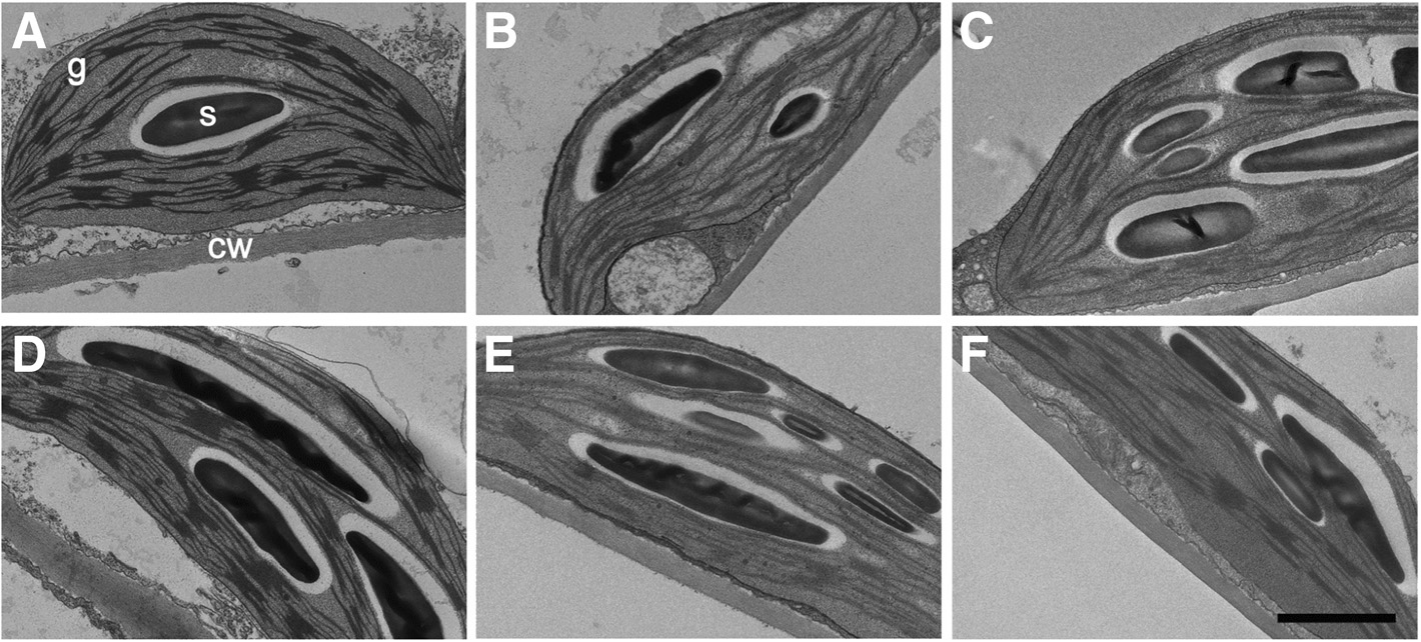
**Transmission electron microscopy of plastids in leaf three of BRZ treated plants.** Plants were grown and harvested as described in Figure 1 at the middle of the light period. **A**, control (0 μM BRZ) at day 3. **B**, 10 μM BRZ at day 3. **C**, 20 μM BRZ at day 3. **D**, control at day 6. **E**, 10 μM BRZ at day 6. **F**, 20 μM BRZ at day 6. Subcellular structures are exemplarily indicated in **A**; cw, cell wall; g, granum (stack of thylakoids); s, starch granule; bar: 1 μm.

Synthetic BR stimulates CO_2_ assimilation [26-29], and *cbb1* and *CPD*-antisense plants exhibit reduced photosynthetic rates [30]. Both biochemical and morphological factors could account for reduced photosynthesis. The consequences of reduced carbon supply include reduced starch levels, impaired energy balance, reduced provision of biosynthetic precursors, and decreased growth [45]. In line with previous reports, starch levels were diminished in *CPD*-antisense, *cbb1*, and BRZ treated plants from day 3 onwards (Figure 8A and B). The reduction of starch levels in *CPD*-antisense and *cbb1* plants is relatively small in comparison to previously determined levels [30]. This may reflect the lack of severe cellular abnormalities that were avoided by the initial BR supplementation. Hexose and sucrose levels were not significantly altered (Additional file 1: Table S1). Examination of plastid ultrastructure revealed intact chloroplasts in BR deficient plants. BRZ treated, *CPD*-antisense, and *cbb1* chloroplasts tended to develop a thylakoid network with reduced grana stacking at day 3 and to a more minor extent at day 6 (Figures 9 and 10).

Early studies on BR demonstrated that inhibitors of protein synthesis (e.g. cycloheximide and puromycin) interfere with BR dependent growth [46]. It was suggested that BR induces the synthesis of a large number of specific proteins, but does not indiscriminately increase overall protein synthesis. In agreement with that view, the overall protein content was not significantly altered in leaves of BR deficient plants (Figure 8C and D).

### Reduced TCA cycle activity in BRZ treated plants

Mitochondrial respiratory metabolism is the major source of ATP and associated with proper maintenance of cellular metabolism as a whole [47,48]. The tricarboxylic acid (TCA) cycle is a crucial component of respiratory metabolism. It links the oxidation of the acetyl group of acetyl-CoA to CO_2_ with the generation of NADH for the oxidation by the mitochondrial respiratory chain. In plants, acetyl-CoA is derived from the products of glycolysis through oxidative decarboxylation of pyruvate by the pyruvate dehydrogenase [49,50].

**Figure 11:**
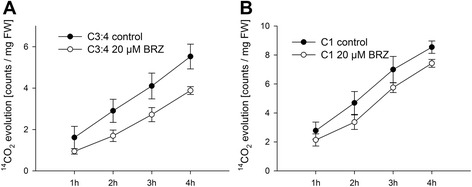
**TCA cycle flux.** Evolution of ^14^CO_2_ of BRZ treated plants at day 6 when incubated with labelled glucose. Plants were grown and harvested as described in Figure 1. **A**, Leaf discs were incubated with [3:4-^14^C]-glucose. **B**, Leaf discs were incubated with [1-^14^C] -glucose. Data are given as mean ± SE (n =5 pools of leaf discs from 10 plants).

Leaf discs were incubated in [3:4-^14^C]-glucose or [1-^14^C]-glucose. CO_2_ from the C3 and C4 positions is preferentially released by the actions of pyruvate dehydrogenase or malic enzyme [51,52]. Feeding with [3:4-^14^C]-glucose to BRZ treated leaves resulted in a lower evolution of ^14^CO_2_ in comparison to the control (Figure 11A). C1 of glucose is released either by an enzyme of the oxidative pentose phosphate pathway (OPPP), namely 6-phosphogluconate dehydrogenase, or an enzyme of the TCA cycle, isocitrate dehydrogenase [51,52]. Feeding of BRZ treated plants with [1-^14^C]-glucose tended to result in a lower ^14^CO_2_ evolution in comparison to mock-treated plants (Figure 11B).

**Figure 12:**
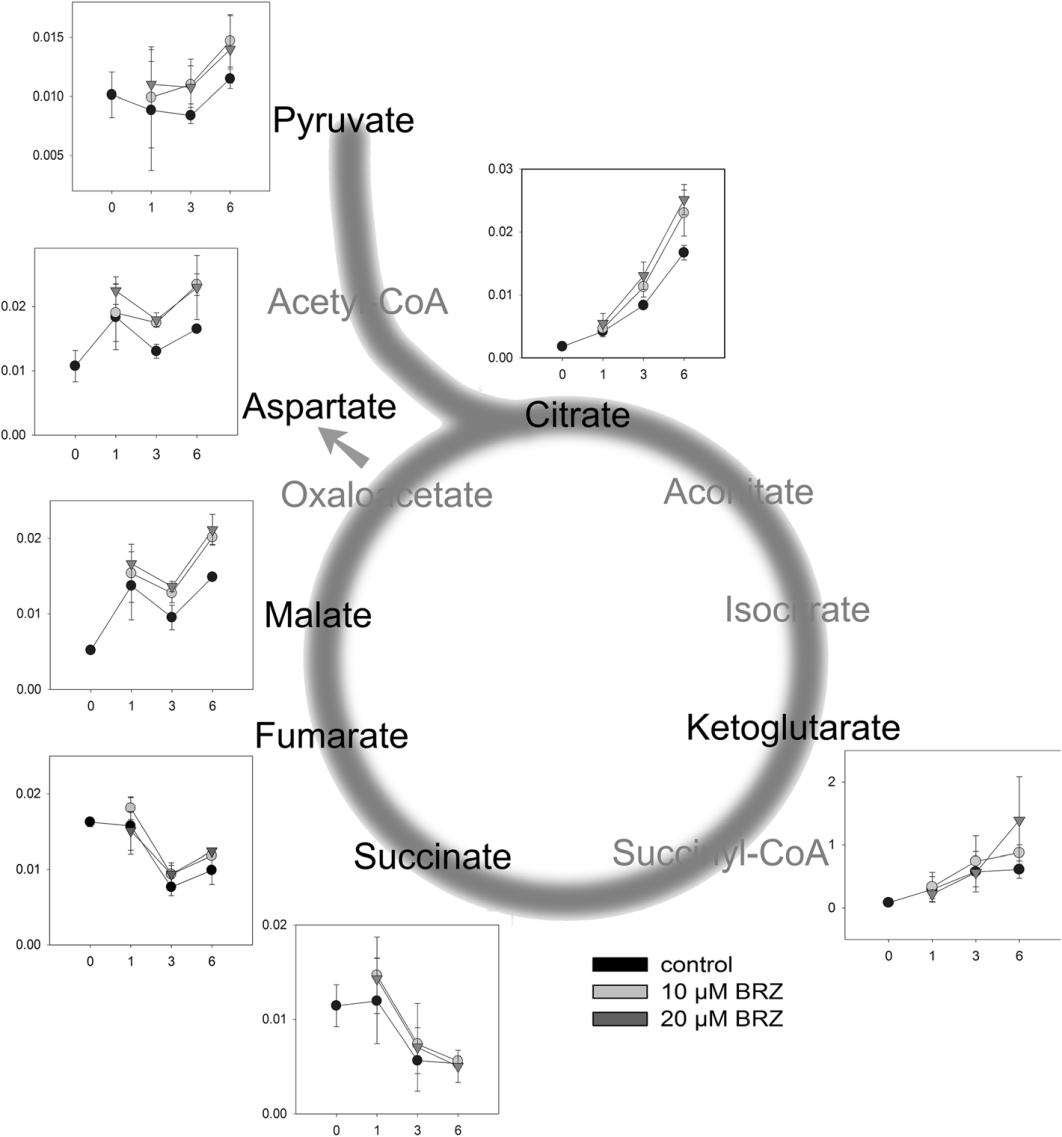
**Levels of TCA cycle intermediates in BRZ treated plants.** Plants were grown and harvested as described in Figure 1. Relative metabolite levels are given as mean ± SE of three biological replicates. Fold change values are given in Additional file 1: Table S2.

The relative content of TCA cycle intermediates was determined by mass spectrometry. Aspartate is synthesized by transamination of oxaloacetate and can be used to estimate oxaloacetate levels. Levels of several TCA cycle intermediates were increased in BRZ treated plants (Figure 12). Levels of citrate, malate, and aspartate were significantly different from the control (Additional file 1: Table S2). A tendency to higher levels of TCA cycle intermediates was also observed in the *cbb1* mutant (Additional file 1: Figure S5). The ketoglutarate level was significantly increased at day 6 (Additional file 1: Table S3).

Lower CO_2_ release from [3:4-^14^C]-glucose by the pyruvate decarboxylase and/or malic enzyme and increased levels of TCA cycle intermediates suggest a weaker TCA cycle activity in BR deficient plants. The release of CO_2_ from [1-^14^C]-glucose is furthermore consistent with a reduced flux through the TCA cycle, but could also suggest a reduced activity of the oxidative pentose phosphate pathway (OPPP).

## Discussion

### Experimental approaches to study BR deficiency

BR deficient plants display dwarfism and multiple defects in cell elongation, cell division, cell differentiation, reproduction and senescence, and light control of development [3]. Reduced fertility and male sterility are common features of BR deficient mutants. BR appears to be largely dispensable for embryogenesis [53]. Seedling development, however, critically depends on BR. Hypocotyl length, cotyledon growth, and responses to environmental stimuli were impaired in Arabidopsis mutants [2,3,12,13]. BR deficiency impairs plant growth at early stages, and later phenotypic changes are inevitably modified by the early growth defects. Thus, mutant analyses can properly address early phases in plant development, but conclusions about BR function at later stages are fraught with uncertainty.

One approach to study the mode of action of BR at later developmental stages is the application of inhibitors of BR biosynthesis [33]. Previous approaches usually supplemented BRZ [33,37,54] and other azole derivatives (e.g. propiconazole, [55]; voriconazole, [56]; YCZ, [57]) to synthetic growth medium, implying that seedlings or small plantlets were analysed. An alternative approach is the complementation of BR deficient mutants for a limited period, and the subsequent deprivation of synthetic BR. Both approaches have pros and cons. For example, BRZ is seen as a highly specific inhibitor, but it presumably also affects other P450s. Although BR deficient mutants respond to synthetic BR, BR feeding cannot fully mimic the endogenous distribution of BR.

For those reasons, both approaches were followed in the current study and analysed in parallel (Figure 1). The first three weeks of the experiments presumed the presence of wild-type BR levels or continuous supply of synthetic BR (mutant complementation for three weeks). At this point (day 0), transcript levels of BR biosynthesis genes and growth were similar in the mutants and the control (Figures 2, 3, 4). Increased *CPD* and *DWF4* transcript levels suggest that plants became impoverished for BR within one to three days (Figure 4).

### BR deficiency in established leaves impairs cell expansion

Leaves grow initially mainly by cell proliferation. Cells divide and grow simultaneously. A proliferation gradient develops between cell division and expansion at the transition zone. The transition from cell proliferation to expansion (cell growth without cell division) is controlled by a network of factors. Cell division first ceases at the tip of the leaf, and progressively ceases along the longitudinal axis [58]. The final size of the organ is achieved by elongation growth.

Mutant analyses indicated that both cell division and cell elongation are affected by BR, because leaves of BR deficient mutant such as *det2* and *cpd* exhibit both decreased cell size and cell numbers [42,44]. BR controls the transition between cell division and expansion [16]. In addition, BR controls organ boundary formation [59,60], xylem formation [61], and stomata development [8,62].

We focused our analysis on established leaves at later developmental stages. At this stage (21 days after sowing and later), cell expansion and cell proliferation took place in leaves three to six. Palisade and spongy parenchyma were significantly smaller in *CPD*-antisense, *cbb1*, and BRZ treated plants (Figures 6, 7, Additional file 1: Figures S1, S2). The cell number was slightly reduced in the *cbb1* mutant (Figure 6, Additional file 1: Figures S1, S3). However, cell proliferation in *CPD*-antisense and BRZ treated plants was similar to the wild type (Figures 6, 7, Additional file 1: Figures S1-3). Thus, cell expansion in established leaves depends on BR, but cell division is barely impaired.

Leaf thickness is determined by the mesophyll anatomy and cell size. The cellular organization of mesophyll tissues modifies the interception of light and CO_2_ diffusion to the sites of photosynthesis. The effects of BR or other phytohormones and stimuli on leaf thickness are not well documented, because changes in leaf growth have usually been assessed in two dimensions. In this study, induced BR deficiency in BRZ treated and *CPD*-antisense plants was associated with reduced leaf thickness (Figure 5, Additional file 1: Figures S1, S2, S4). In contrast, leaf thickness in *cbb1* plants increased more until day 6 (Figure 5, Additional file 1: Figures S1, S4). The reason for the difference between the genotypes could be the incomplete normalization of *cbb1* plants. Alternatively, *cbb1* plants could synthesize alternative BR and respond in a different manner to BR as has been reported for the rice *brd2* (*BR-deficient dwarf2*) mutant (Hong *et al.* [35]).

### Reduced starch accumulation may cause reduced growth

Reduced growth of BR deficient plants may be a consequence of reduced carbon availability. Starch levels in *cbb1*, *CPD*-antisense, and BRZ treated leaves were lower in comparison to the wild type (Figure 8). This presumably is a consequence of drastically reduced CO_2_ assimilation rates [30]. Optimal starch metabolism is pivotal for the diurnal carbon balance and growth [45]. Mutants impaired in starch synthesis such as *phosphoglucomutase (pgm*) or mutants impaired in starch degradation such as *starch excess 1* (*sex1*) show dwarfism [63]. Both too rapid and too slow mobilization of starch during the night can result in diminished growth rates [64]. Thus, carbon undersupply may cause impaired growth in BR deficient mutants.

Electron micrographs of BRZ treated plants and the *cbb1* mutant revealed a tendency to less grana thylakoids (Figures 9, 10). The molecular basis of reduced thylakoid stacking is unknown. It could reflect a delay in plastid development, an altered adaptation of the thylakoid architecture to the light conditions, an altered protein composition, or changes in other regulatory mechanisms. Grana confer functional advantages such as enhancement of light capture and fine-tuning of energy distribution between the photosystems [65,66]. Conceivably, the observed changes in chloroplast structure contribute to the reduced photosynthetic rate and starch accumulation. Plastid structure and function were previously analysed in BR mutants. One reason for that interest is the link between BR action and photomorphogenesis [12]. Light-grown *det2* plants developed structurally altered chloroplasts. For example, eight-day-old *det2* chloroplasts had a smaller, rounder shape, reduced grana stacking, and an abnormally high ratio of chlorophyll a/b in comparison to the wild type, indicating an immature status [67]. However, Azpiroz and coworkers [68] did not describe an altered chloroplast structure of light-grown *dwf4* plants. Given the multiple reports that describe altered properties of plastids of BR treated plants or BR mutants ([69] and references therein), a more detailed analysis of the underlying structural and molecular changes may be worthwhile.

### Reduced growth is associated with reduced TCA cycle activity

The TCA cycle links the oxidation of pyruvate and malate with the generation of NADH. NADH is used by the mitochondrial respiratory chain for ATP production. The reduced release of ^14^CO_2_ from labelled glucose in BRZ treated plants (Figure 11) and elevated levels of TCA cycle intermediates in BRZ treated (Figure 12) and *cbb1* plants (Additional file 1: Figure S5) suggest a reduced carbon flux through the TCA cycle in BR deficient plants. Reduced TCA cycle activity may compromise efficient use of carbohydrates and impair growth especially during the dark period. Furthermore, the TCA cycle provides precursors for various biosynthetic pathways [48,50].

Reduced production of ATP for sucrose synthesis and carbon precursors for anabolism could be consequence or cause of reduced growth. BR deficiency and reduced growth presumably goes along with reduced demand for carbohydrates, amino acids, and other biosynthetic precursors. On the other hand, reduced photosynthesis in BR deficient plants could diminish the supply of substrates for mitochondrial reactions and reduce the flux through the TCA cycle. The situation becomes even more complex in view of the multifaceted links between photosynthesis and TCA cycle. Altered TCA cycle enzyme activities can result in increased, decreased, or unvaried photosynthesis [70,71]. Thus, identification of cause and effect of metabolic changes is complicated. Labelling studies and application of network models will be necessary to precisely determine the flux of metabolites and interplay of metabolic pathways in BR deficient plants.

## Conclusions

The morphology of BR deficient mutants was described in detail. Numerous studies addressed the consequences of BR deficiency at the molecular and cellular level. In that way, the current understanding of BR was developed. However, the analysis of BR deficient mutants is complicated by the dwarfism and multiple morphological changes. The mode of action of BR at later developmental stages cannot be faultlessly determined. In this study, we used two approaches for the analysis of BR action in established leaves. An inhibitor of BR biosynthesis was applied and BR complementation of BR deficient plants was stopped after three weeks. For the first time the metabolic changes upon BR deficiency were analysed comprehensively by means of metabolic profiling. Our analyses revealed that induced BR deficiency impairs starch accumulation, TCA cycle activity, cell expansion, and biomass production. Further studies are needed to determine alterations in metabolic fluxes and the precise link between genomic BR effects and catabolic and anabolic pathways. Transgenic approaches such as the inducible expression of RNAi hairpins represent another approach that would enable tissue-specific repression of BR biosynthesis. This could particularly help to separate the role of BR in sink and source tissues.

## Methods

### Growth conditions

C24 wild type was obtained from the Nottingham Arabidopsis Stock Centre (NASC) - NASC ID: N906. The *CPD*-antisense line and the *cbb1* mutant were described before [13,30]. Seeds for growth experiments were derived from plants grown in parallel in a greenhouse. The *cbb1* mutant was repeatedly treated with BR before and during seed set. Seeds were allowed to germinate and seedlings grew for two weeks in controlled growth chambers (7 days: 16 h light [140 μmol m^−2^ s^−1^, 20°C, 75% relative humidity]/8 h night [6°C, 75% relative humidity]; thereafter 7 days: 8 h light [140 μmol m^−2^ s^−1^, 20°C, 60% relative humidity]/16 h night [16°C, 75% relative humidity]). Subsequently, plants were transferred to long-day conditions in a controlled growth chamber (16 h light [140 μmol m^−2^ s^−1^, 20°C, 60% relative humidity]/8 h night [16°C, 75% relative humidity]). All genotypes were grown in the same chamber at the same time in a randomized manner, each replicate one after another. All necessary measures were taken in order to avoid biotic and abiotic stress.

Plants were sprayed at midday for three (BL) or five (BRZ) times a week with an aqueous solution containing BL or BRZ, respectively, and 0.01% Tween 20. Methanol was used as solvent for stock solutions. The same volume of methanol was added to the control solution. BL and BRZ experiments were performed in the same growth chamber. BRZ was sprayed more often to ensure lowered BR levels.

### Gene expression analysis

Gene expression analyses were performed as described before [72]. Primer sequences for quantitative RT-PCR were as follows: CPD_fw 5’ GGA AAC ACT CTC TGC TTC TTA TGA AAG GT 3’, CPD_rev 5’ AAG TAA AGC CAC CAA GAA GTC AAC AAT CT 3’, DWF4_fw 5’ AAT CCT TGG AGA TGG CAA CAG C 3’, DWF4_rev 5’ TCT GAA CCA GCA CAT AGC CTT GG 3’, eIF1α_fw 5’ TTG ACA GGC GTT CTG GTA AGG 3’ and eIF1α_rev 5’CAG CGT CAC CAT TCT TCA AAA A 3’ (At5g60390).

### Microscopy

Light microscopy was performed as described before [72]. Cell size and number determination covered all parts of the leaf sparing cells surrounding the primary vein and at the edge of the leaves. For transmission electron microscopy, leaf samples were fixed in 2.5% glutaraldehyde, 0.1 M cacodylate buffer (pH 7.4), 5 mM calcium chloride for 4 h at 4°C, and post-fixed with 1% Os0_4_ and 0.8% K_3_Fe(CN)_6_ for 2 h at 4°C. The samples were washed with water and post-stained with 2% aqueous uranyl acetate for 2 h. Subsequently, the tissue was dehydrated in a series of ethanol and propylene oxide and embedded in Spurr’s low viscosity epoxy resin. Ultrathin sections (60–70 nm) were cut with a Leica UC6 ultramicrotome using a diamond knife, stained with uranyl acetate and lead citrate and examined on an energy-filtering transmission electron microscope (EFTEM, Zeiss) at 120 kV.

### Protein and starch levels

Protein and starch levels were determined in leaves one to four. The Quick Start™ Bradford Protein Assay (BioRad) was used as described in the manufacturer’s description. Starch levels were determined as described before [73].

### Metabolite analysis

50 mg of powdered plant material from leaves one to four was used per extraction. Extraction of metabolites, LC-MS measurements and data analysis was performed as described by Giavalisco and coworkers [74]. For GC-MS analysis, the polar phase of the same extraction was used and carried out as described before [75].

### ^14^C-flux analysis

Leaf discs from leaves three and four were incubated with labeled glucose in the light. Capture of the released CO_2_ and analysis of the samples were performed as described before [51].

### Availability of supporting data

The data sets supporting the results of this article are included within the article and its additional file.

## Additional file

Additional file 1: Figure S1. Growth parameters of rosette leaves five and six of *CPD*-antisense and *cbb1* plants. **Figure S2.** Growth parameters of rosette leaves five and six of BRZ treated plants. **Figure S3.** Epidermis cell number of leaves three and four. **Figure S4.** Transversal sections of rosette leaves three and four. **Figure S5.** Relative levels of TCA cycle intermediates in the wild type and *cbb1* mutant. **Table S1.** Relative hexose and sucrose levels. **Table S2.** Relative levels of TCA cycle intermediates in BRZ treated plants. **Table S3.** Relative α-ketoglutarate levels in the *cbb1* mutant.

## Abbreviations

*aCPD*: *CPD-antisense*
BL: Brassinolide
BR: Brassinosteroid
BRZ: Brassinazole
GC-MS: Gas chromatography–mass spectrometry
LC-MS: Liquid chromatography-mass spectrometry
OPPP: Oxidative pentosephosphate pathway
TCA: Tricarboxylic acid
