# Supplementary material for: Consequences of induced brassinosteroid deficiency in Arabidopsis leaves
Source: BMC Plant Biol. 2014 Nov 18;14:309. doi: 10.1186/s12870-014-0309-0 (PMC4240805; doi:10.1186/s12870-014-0309-0)
Supplement: Additional file 1: Figure S1. — Growth parameters of rosette leaves five and six of CPD-antisense and cbb1 plants. Figure S2. Growth parameters of rosette leaves five and six of BRZ treated plants. Figure S3. Epidermis cell number of leaves three and four. Figure S4. Transversal sections of rosette leaves three and four. Figure S5. Relative levels of TCA cycle intermediates in the wild type and cbb1 mutant. Table S1. Relative hexose and sucrose levels. Table S2. Relative levels of TCA cycle intermediates in BRZ treated plants. Table S3. Relative α-ketoglutarate levels in the cbb1 mutant. [file 12870_2014_309_MOESM1_ESM.pdf]

Consequences of induced brassinosteroid deficiency in Arabidopsis leaves

Florian Schröder, Janina Lisso, Toshihiro Obata, Alexander Erban, Eugenia Maximova, Patrick Giavalisco, Joachim Kopka, Alisdair R. Fernie, Lothar Willmitzer, Carsten Müssig

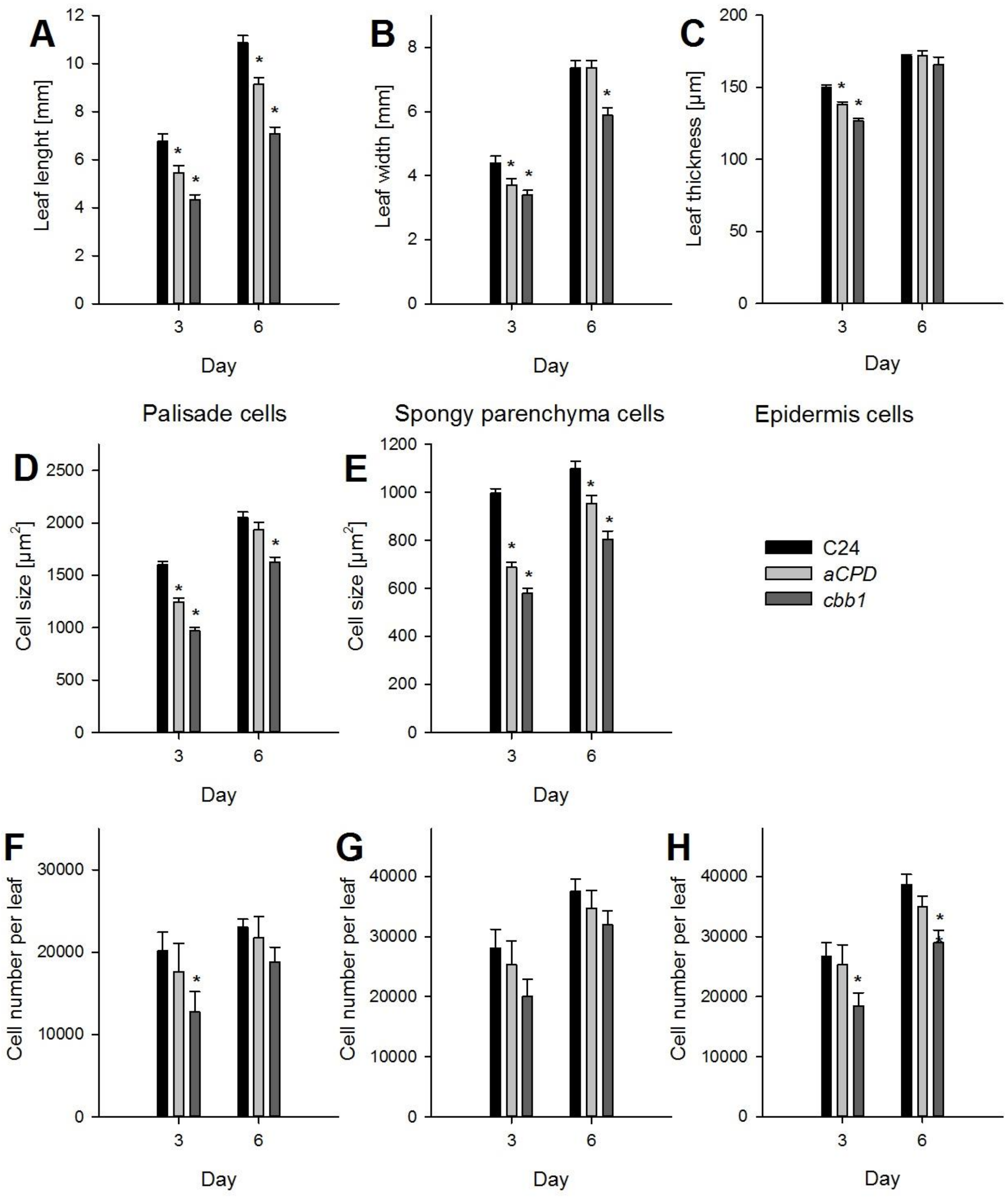

Figure S1. Growth parameters of rosette leaves five and six of *CPD*-antisense and *cbb1* plants.

Leaves were analyzed as described in Figure 2 and Figure 5. Data are given as mean ± SE. 20 leaves were analysed per point in time. Values denoted with an asterisk are significantly different from their control (t test, $P < 0.05$). A, Leaf length. B, Leaf width. C, Leaf thickness. D, Area of palisade cells. E, Area of spongy parenchyma cells. F, Number of palisade cells per leaf. G, Number of spongy parenchyma cells per leaf. H, Number of epidermis cells per leaf.

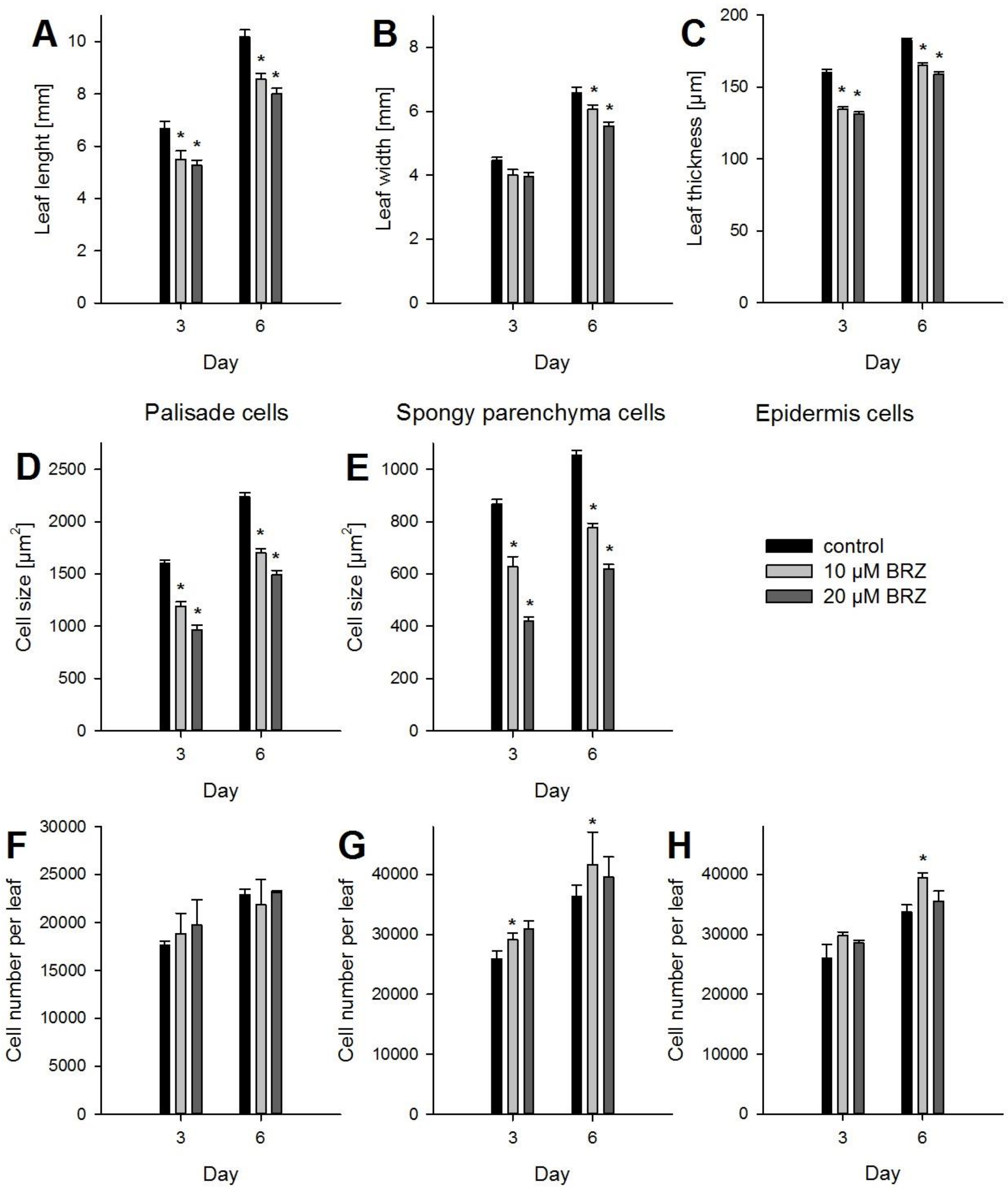

Figure S2. Growth parameters of rosette leaves five and six of BRZ treated plants.

Leaves were analyzed as described in Figure 3 and Figure 5. Data are given as mean ± SE. 20 leaves were analysed per point in time. Values denoted with an asterisk are significantly different from their control (t test, $P < 0.05$). A, Leaf length. B, Leaf width. C, Leaf thickness. D, Area of palisade cells. E, Area of spongy parenchyma cells. F, Number of palisade cells per leaf. G, Number of spongy parenchyma cells per leaf. H, Number of epidermis cells per leaf.

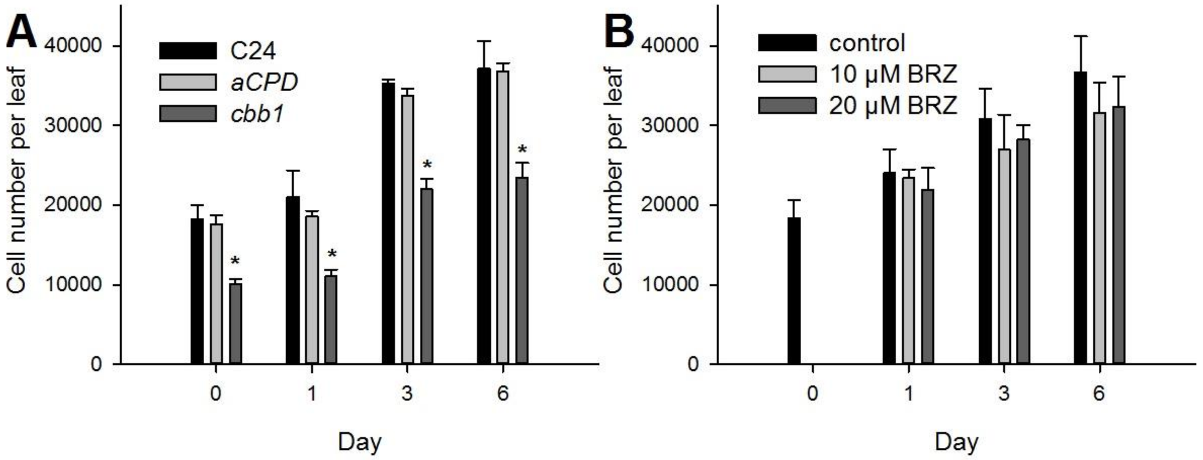

Figure S3. Epidermis cell number of leaves three and four.

Cell numbers were calculated from leaves bleached in 1 M KOH. Data are given as mean ± SE. 20 leaves were analysed per point in time. Values denoted with an asterisk are significantly different from the wild type or control (t test, $P < 0.05$). A, Epidermis cell number of wild-type (C24), *CPD*-antisense, and *cbb1* plants. B, Epidermis cell number of BRZ-treated plants.

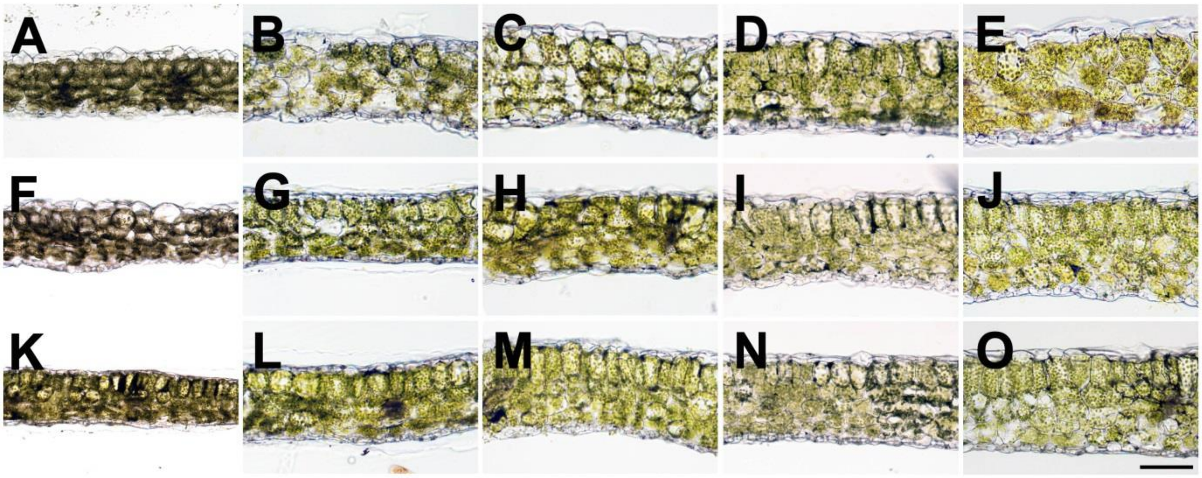

Figure S4. Transversal sections of rosette leaves three and four.

Leaves 3 and 4 were embedded in 4% agarose and sectioned through the widest part of the blade. A, Wild type (C24) at day 0. B, C24 at day 3. C, C24 at day 6. D, control (0 µM BRZ) at day 3. E, control at day 6. F, *CPD*-antisense at day 0. G, *CPD*-antisense at day 3. H, *CPD*-antisense at day 6. I, 10 µM BRZ at day 3. J, 10 µM BRZ at day 6. K, *cbb1* at day 0. L, *cbb1* at day 3. M, *cbb1* at day 6. N, 20 µM BRZ at day 3. O, 20 µM BRZ at day 6. Bar represents 100 µm.

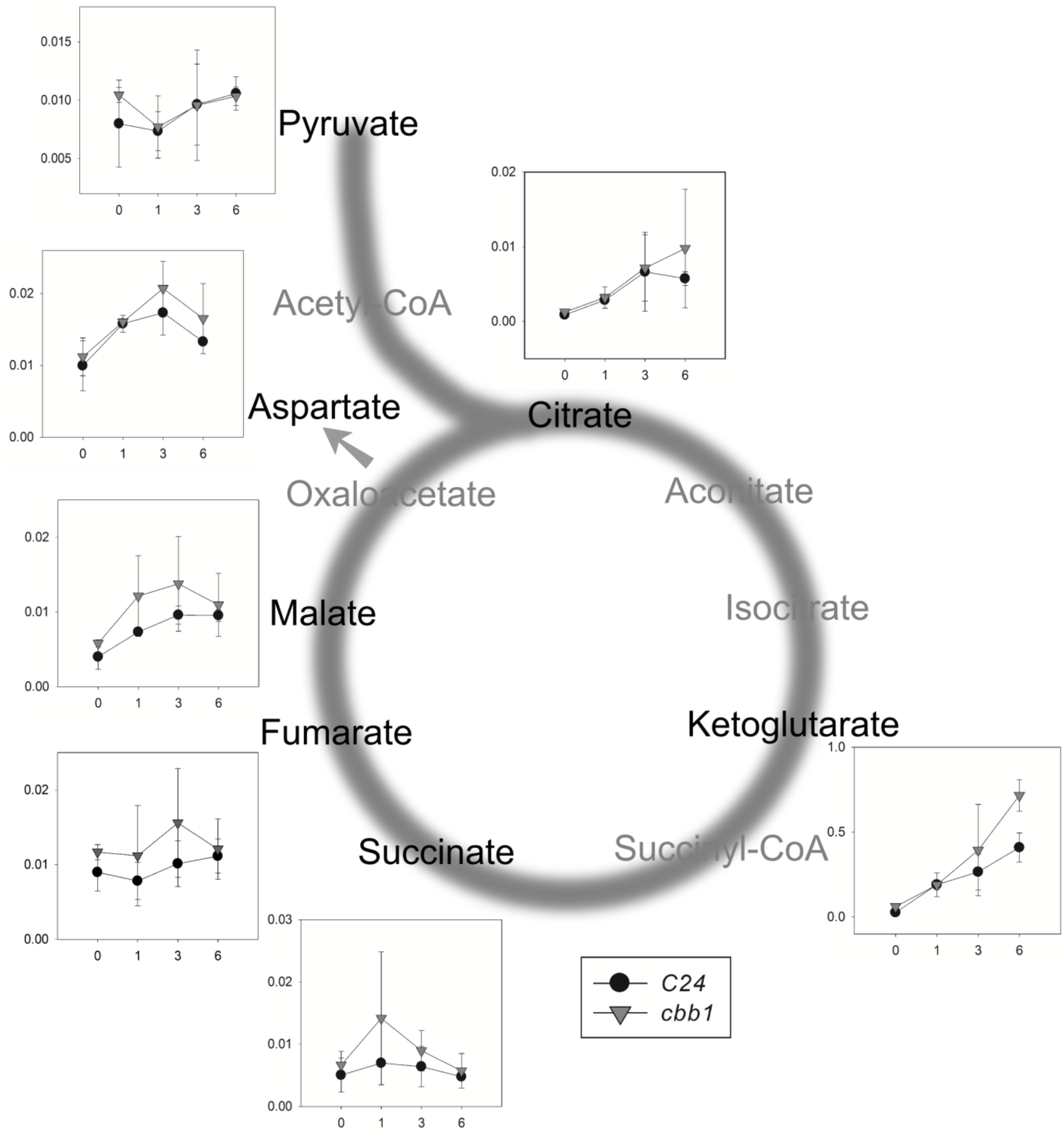

Figure S5. Relative levels of TCA cycle intermediates in the wild type and *cbb1* mutant.

Plants were grown and harvested as described in Figure 1. Relative metabolite levels are given as mean ± SE of three biological replicates. Fold change values of significantly changed metabolites are given in Table S3.

Table S1. Relative hexose and sucrose levels.
Values are given as fold change (BR deficient plant vs control). Values are given in bold if metabolite levels were significantly different (t test, P < 0.05).

Metabolite	10 µM BRZ/control			20 µM BRZ/control			*cbb1*/wild type			
	day 1	day 3	day 6	day 1	day 3	day 6	day 0	day 1	day 3	day 6
Glucose	1.13	**1.22**	0.96	0.95	1.21	1.01	1.37	0.99	1.19	1.66
Fructose	1.47	**1.31**	1.01	0.96	1.10	1.27	1.83	2.12	1.83	2.94
Sucrose	1.05	1.09	1.13	0.99	1.04	1.17	1.52	0.95	1.13	0.98
Glucose-6-phosphate	1.01	1.07	1.13	0.85	1.25	1.12	1.37	1.11	1.02	1.10
Fructose-6-phosphate	1.01	1.26	1.16	0.84	1.49	1.19	1.36	1.02	1.14	0.99

Table S2. Relative levels of TCA cycle intermediates in BRZ treated plants.
Values are given as fold change (BR deficient plant vs control) and in bold if metabolite levels were significantly different (t test, P < 0.05).

Metabolite	10 µM BRZ/control			20 µM BRZ/control		
	day 1	day 3	day 6	day 1	day 3	day 6
Aspartic acid	1.04	**1.34**	**1.42**	1.22	**1.38**	1.39
Citric acid	1.11	**1.35**	1.38	1.30	**1.56**	1.51
Malic acid	1.12	1.34	**1.36**	1.21	**1.43**	**1.42**

Table S3. Relative α-ketoglutarate levels in the *cbb1* mutant.
Values are given as fold change (BR deficient plant vs control) and in bold if metabolite levels were significantly different (t test, P < 0.05).

Metabolite	*cbb1*/wild type			
	day 0	day 1	day 3	day 6
α-Ketoglutarate	2.28	0.99	1.48	**1.75**
